# Supplementary material for: Functional Analysis of Steroidogenic Factor 1 (sf-1) and 17α-Hydroxylase/Lyase (cyp17α) Promoters in Yellow Catfish Pelteobagrus fulvidraco
Source: Int J Mol Sci. 2020 Dec 27;22(1):195. doi: 10.3390/ijms22010195 (PMC7795741; doi:10.3390/ijms22010195)
Supplement: Supplementary file 1 [file ijms-22-00195-s001.pdf]

Table S1. Primers used for *sf-1* and *cyp17α* promoters cloning and 5’-deletion plasmids construction in yellow catfish.

Gene	Primers	Forward primer (5'-3')	Reverse primer (5'-3')
sf-1	Outer primer (TSS)	GCTGATGGCGATGAATGAACACTG	ATTTGAAAGTGCTTGCTCGGA
	Inner primer (TSS)	CGCGGATCCGAACACTGCGTTTGCTGGCTTTGATG	GCCAAACTTGTTCCTCCCT
	pGl3-489/+254	ctatcgataggtaccgagctcTCCCTTGTGATTCCATAAGATCAG	cagtaccggaatgccaagcttCCTTTACTTTGCGCCTCCAG
	pGl3-921/+254	ctatcgataggtaccgagctcGCGTAGATTTTAACATCATCATTGC	cagtaccggaatgccaagcttCCTTTACTTTGCGCCTCCAG
	pGl3-1421/+254	ctatcgataggtaccgagctcAATTCATGTCTATGACGTGGTGAATT	cagtaccggaatgccaagcttCCTTTACTTTGCGCCTCCAG
	pGl3-1981/+254	ctatcgataggtaccgagctcTGTTCATCCAGCACTAAACACTGTAG	cagtaccggaatgccaagcttCCTTTACTTTGCGCCTCCAG
cyp17α	Outer primer (TSS)	GCTGATGGCGATGAATGAACACTG	AATACAATGGTCTGGAGCTT
	Inner primer (TSS)	CGCGGATCCGAACACTGCGTTTGCTGGCTTTGATG	TTCCCTCCACGAGTCAGCA
	pGl3-330/+68	ctatcgataggtaccgagctcACATATATTAAGGAAAAAAAGAAAGTTCTAGA	cagtaccggaatgccaagcttGTCTCTGGGAAGCTCATTAGAGCC
	pGl3-687/+68	ctatcgataggtaccgagctcTGTAGAGGAATAACATACGGTCATACAT	cagtaccggaatgccaagcttGTCTCTGGGAAGCTCATTAGAGCC
	pGl3-1402/+68	ctatcgataggtaccgagctcCAACATACAGCAATCTTGTTCTGTCA	cagtaccggaatgccaagcttGTCTCTGGGAAGCTCATTAGAGCC
	pGl3-2034/+68	ctatcgataggtaccgagctcGTTAGGTTGGCTTGCTTATGATAAGA	cagtaccggaatgccaagcttGTCTCTGGGAAGCTCATTAGAGCC

Table S2. Primers used for overexpression plasmids of yellow catfish PPARα, PPARγ and STAT3 into pcDNA3.1 (+) vector.

Genes	Forward primer (5'-3')	Reverse primer (5'-3')
PPARα	ctagcgtttaaacttaagcttATGGCTGAATTATTTTTAAAACAGACC	aacgggccctctagactcgagTTACTTATCGTCGTCATCCTTGTAATC
PPARγ	ctagcgtttaaacttaagcttATGGTGGACACACAGACGTTTTC	aacgggccctctagactcgagCTACTTATCGTCGTCATCCTTGTAATC
STAT3	ctagcgtttaaacttaagcttATGGCCCAGTGGAATCAGTTG	aacgggccctctagactcgagTCACTTATCGTCGTCATCCTTGTAA

Table S3. Primers used for site-mutation analysis.

Gene	Primers	Forward primer (5'-3')	Reverse primer (5'-3')
sf-1	Mut- *sf-1*-PPARα	TGgcggcctgtaTAGTTTTTGTTTCTTTTCCGCACA	AAACTAtacaggccgcCAGGACTAAAATGTATTATTTTTATTTATTTATTT
	Mut- *sf-1*-PPARγ	GAATcagtcggcaTAGAGTTAATTTAACTGAACGCTGCTG	TCTAtgccgactgATTCACCACGTCATAGACATGAATTA
	Mut- *sf-1*-STAT3	AACAagcggatcggcTACAAAAATAGAAGCAAAGCCGG	TAgccgatccgctTGTTTATTATTTTGTGCCATAGTGAAA
cyp17α	Mut-*cyp17α*-PPARα	cgagttacagcggacttgCACTGCTCACAAACCAGAGACTGA	caagtccgctgtaactcgGCTCATGACTATCAATGCATTTGTG
	Mut-*cyp17α*-PPARγ	tacgactacgccgtaatcgcTGAAGGCTGTGGGAATGTCC	gattacggcgtagtcgtaAGAAACTGATGGTCATGAAACTGATAA
	Mut-*cyp17α*-STAT3	GAAAAgctactcgcgTGAATCCTGATGTAAACACTGTGCC	TCAcgcgagtagcTTTTCCACTGCATCTGTCAAGC

Table S4. Primers used for electrophoretic mobility-shift assays.

Primers		Forward primer (5'-3')	Reverse primer (5'-3')
sf-1-PPARα	Biotin-probe	Biotin-CCTGTATTTTCACCTAGTTT	Biotin-AAACTAGGTGAAAATACAGG
	Mutative-competitor	CCTGGCGGCCTGTATAGTTT	AAACTATACAGGCCGCCAGG

sf-1-PPARγ	Biotin-probe	Biotin-GTGAATTGACTTTTGTAGAGT	Biotin-ACTCTACAAAAGTCAATTCAC
	Mutative-competitor	GTGAATCAGTCGGCATAGAGT	ACTCTATGCCGACTGATTCAC
sf-1-STAT3	Biotin-probe	Biotin-TAAACACTTAAGGAAAATACAAA	Biotin-TTTGTATTTTCCTTAAGTGTTTA
	Mutative-competitor	TAAACAAGCGGATCGGCTACAAA	TTTGTAGCCGATCCGCTTGTTTA
cyp17α-PPARα	Biotin-probe	Biotin-GCATGTGCGTCAAAAGTCCACA	Biotin-TGTGGACTTTTGACGCACATGC
	Mutative-competitor	GCCGAGTTACAGCGGACTTGCA	TGCAAGTCCGCTGTAACTCGGC
cyp17α-PPARγ	Biotin-probe	Biotin-CCATGACCTTTAACCCCTAA	Biotin-TTAGGGGTTAAAGGTCATGG
	Mutative-competitor	TACGACTACGCCGTAATCGC	GCGATTACGGCGTAGTCGTA
cyp17α-STAT3	Biotin-Probe	Biotin-GGAAAATTCCAGAAAATGAATC	Biotin-GATTCATTTTCTGGAATTTTCC
	Mutative-competitor	GGAAAAGCTACTCGCGTGAATC	GATTCACGCGAGTAGCTTTTCC

```
TGTTCATCCA GCACTAAACA CTGTAGTCAT CAACTCAAGA CGTGACGTCA TCATTGTTTA GGACTTTAGT GAACACTGCA AGTGTTAATT CAACAATTTA GGAATCTCCA GAGTTAAAAT -1862
                                            CREB1
GAACTCTATG GACATTCAAG AGTTAAACCA TTGCAAATGT GTTAATTCCT CTGTCCTAAC ATTGTTACTA AATCAACACA AGAGTTAACA CTATCAAAGC AAATCCAGCT CTGTGTGTGT -1742
TAATTACACC GTGCTAATAC AACAACGAAA TATCTGAGAG TACTGTAGGT GTTTGTTCAC AATAATGAAC ACTACATGTG TTAAGTTGAG GAAAAAGTAG ATTCAACGAC CTATGGGTTA -1622
ATTAAACGAC CCTTAACTAA CAATATACAA GTTATTACAG TGCTGTATGT TATGTAACAC TTTAGGTGTT AATTCAGCAG TGTTGAATCA AACTCAATGC AGTGTGTTCG ATTTAATCTG -1502
                                                                                                          CREB3
TCCTTATTAC TAATGCAGTT CAACCAATGG ATGCTCACTA GAGTTAATTC AGCTCTGTAA AGTCTGCAAA TCTATGTGTT AATTCATGTC TATGACGTGG TGAATTGACT TTTGTAGAGT -1382
                                                                                                    SREBF1       PPARγ
TAATTTAACT GAACGCTGCT GTATATTAAA GCTACATACA TTTTAGTATT TATTTTATTT ATTCCTTTAG TATTCTGTAT GTGAAGACTA ATGGTCTTTA GCATGTCCAC ACCCTGTGGT -1262
TTATTGACCA TAAATACACA GAAACACTGC AAATTGTTTT GTTTTCTTAA TATTATTTCT TTAACTGCTT TAAACTTTTT GAACTGAATT AGTTTTTACA TGTTTTTACA ATGGGAAATG -1142
TAGTTTTATT TCACTATGGC ACAAAATAAT AAACACTTAA GGAAAATACA AAAATAGAAG CAAAGCCGGT TATGAAGTTT GGAAAGTTTA GAAACTTCTA CTTTGTTTAA AAGCTAAAAT -1022
                                    STAT3
CATCCAAATA TTCACAAAAA ATGTGTAACG TCGCGTTTAT AAAATGGTCG ATTTTTTTTT CTTTTCAACA CATTTAGCGT AGATTTTAAC ATCATCATTG CATCATTCTT TTACGTCTGA -902
                                                                                                         C/EBPß
AAATCTGAAA AAGGGTTTTG AGACTTTTGA AATTTAAAAG TCAAACATTT TCACATTTTT CATCTGAAGT TTGAAGGCCA TGTGAATGTT TTATTTTTAG ATTTTGTTTT AAATTCTTTT -782
                         C/EBPα
TTTCCCCTTT TTTTTTATTA ATGTGTGATT TTCAATATTT TTTCCTTATT TCTTTCACAT ATTTAAAAAA ATTGTTAGAG AAGAAGAAAA ACATCTCAGT TAATTTCATG TCAATGTCTG -662
AAGCCAGTGT GTGTGTTTGT GTGTGTGTGT GTGTGTGTGT GTGTGTGTGT GTGTGTGTGT GTGTGTGTGT GTGTGTGTGT GTGTGTGTGT GTGTGTGGTT TCTTCTCTTT CTTTCATTTT -542
TATTTGGTGT AAATACGGGG ATTTATTTAA TTATATAATT TACAATATTA TTTCCCTTGT GATTCCATAA GATCAGATGG AAACAGATGG AATAATAAAT AAATAAATAA AAATAATACA -422
                                  CREB5
TTTTAGTCCT GTATTTTCAC CTAGTTTTTG TTTCTTTTCC GCACATTTTT TTAGACTTTA ATATTTGGTG ATAAAGCTGC ATAACACATC ATTCATTACA GATTTCTCAG ACCTCCATAA -302
                PPARα                                                                          SREBF2
CGTGCCTATT TATTTATTCA CCAATTTATT TTGTGCAGCA CAAACATCTT TGTTCATTTT GTTGCAGTGT TGTAGAGCGT TGAAGCAAAC CGTCAGTTGG ACAAAAATGT TCACGTGTTA -182
ATTCAATACT GTGGTGTTCA TTCAAATCTG TAGAATTGGG CAGAAGCGCA TCCTCCTCCT CCTCCTCCTC CTCCTCCTCC TCCTTCTCAT CTCCAGTGTA TGTGTGAGTG TGTAAATGTG -62
                                                          SP1         SP1
TTTAGCGAGT CACATGAGTG TGTGATGTCC AATGAAGGCG CCTCTCCTCT CCTCCCGGCG CATAAAGCGG ACTCACTCAC ACACACTCAA CATGGGCGAG CGGGGACGGT GCAGCAGCTG +59
                            CCAAT box                SP1          TSS
AACACCATCT CACACAACTT CACACACTCC ATCTCCATCT CCAACTCGTG TGCGCGCGCG CGCGTGTGTG TATATGTATG TATCTATGTG CGTGTGTCTG TTTGGACACG TCCGGAGCTG +179
CTGAGGAGCG TCGCCGTCTT TTTCCTTATA CACACAACAC ACACTGAGTC CAATGCTGGA GGCGCAAAGT AAAGG +254
```

Figure S1. Nucleotide sequence of yellow catfish *sf-1* promoter. Numbers are relative to the transcription start site (+1). The putative transcription factor binding sites are underlined. The highlighted sequences show putative transcription factor binding sites.

```
GTTAGGTTGG CTTGCTTATG ATAAGACTAT TTACAGTAGC AATCCAAAAT GTATGTACCT TAACATTAAA TGTGAATATA TTTCTGCAAG CTTTTAAACT -1935
TTTATACCAA AAATACAAAT CTGCTTTAAG TCAACTCAGG CCATATTTCC CCCTTTATTA ACATTAGTTC ACTTTTTTTT TTTCTTTAGG TGGCTAAACA -1835
GCAAAATTAC AAAAATGTTC CTACAAAACA TCTAATACAG AAATTGATTT TTTTTTTTTT TTACTTTAAA TTGCTGATTT AAGCACAAAA ACCGTCAATG -1735
CCTGCACTTG ATTTCTTTGC CTTTTCTGCT TTGGTGCTCT GTAGCTTGAC AGATGCAGTG GAAAATTCCA GAAAATGAAT CCTGATGTAA ACACTGTGCC -1635
                                                                       STAT3
TGTTCCCTTT CAGGTCCAAC TTCACGAGCC AGCATGTAGA GTTATTCGTT ATCAATCCAG TTTGGGTCAC ATGATCCCAA CTTCCTGACC CAACAAGCTA -1535
                 CREB1                                                 SREBF1
AACCCAGCAA CTGAATTATA AAATTAGACC TCACATAATT ATATATAGTT AACATCATTT GGGGCTTCTA AGTAATAATG ATAATGGTAA CATTACATCT -1435
AGTTAACAAT TTTATTGCAT TTAATTCTGT AACAACATAC AGCAATCTTG TTCTGTCAGC CATATTCGAA GATGTTTGTT CACTCTGTGC GGACCAGATG -1335
AAGGTGCGTG TATGGATTGT TGCTGGTCCA TCTTTAATTT TATATCTGAA ATTAGTCATT AATGGACAGG ATTAGAGTTT TGAGCAGCAG AAAGATGATC -1235
GTAGCTCAGC CTCATGCTGC TAGAAGAATA TGAGCGCACA TAGAGACACT GTATGTCTGT CCTCCTCCAT GTGAACATCT GAGTCTCTCT GACACACTGG -1135
GTTATAATTA GAGCTGAAAC ATTCGTCTTA CAGACCTGTT TCTCTCTCTG TCCGCAGTGT ATGTGTGCAT GTGGTGTGTG TGTGTGTGTG TGTGTGTGTG -1035
TGTGTGTGTG TGTGTGTGTG TGTGTGTGTG TGTGTGTGTG TGTGTGTGTG TGTGTGTGTG TGTGTGCGTG TGCGTGTGTG TGAGTCCTTG GCTAATTTAC -935
ATGGCCTTGA CTAAGAAAGC GAGTCTGGCA CAAATGCATT GATAGTCATG AGCATGTGCG TCAAAAGTCC ACACTGCTCA CAAACCAGAG ACTGAAGAGC -835
                                                             PPARα
TTGTTATAAA AAGCAGATGG AGGAACAGGT GACAAAAATT TATTTTAAAA AAAAATAGAC ATTTTAAAAA GATGAAACTC ATTAAGCAGA ATGGATTTGT -735
TAAATATAAC CCGTGCTATA TTATCTTCCG ATATGAACAG GTTTACATGT AGAGGAATAA CATACGGTCA TACATACATT CTAAAAAAAT AAAGACCTAG -635
AACATAAACA GTATTTTATA AGGATATTTT ATCATTTATA AAGTCTTTCT GTACTCACAG TGGTATGACT GATATAAAGC AGATGAACTC AATATATTTA -535
                                                              SREBF2
TTTAGATATA ATGTGGCTCA GATCCAGAAT CAGATTTAAA TGTTGATAGG GAAAATATAT AAAATCAGAG TAAAGAGTTT AATTAACAAA TCTGTATATA -435
ACTAAGTTCT AAAAAAAAAA AAAAAAATAC TGTTACGTAT TTTGCCGTAA GATATCAACA AATACTCTTT CTTTTGTGGT GTAACATGTA TATTTTGTGG -335
                                   CREB5                                     C/EBPß
TGTTACATAT ATTAAGGAAA AAAAGAAAGT TCTAGAGTGA AAAGATTCAT TGTACAAGAT TTTAAATATA AATTCATATT TATTTTTTGT TTTGAGAATT -235
                                                C/EBPα                                        TATA box
TTTCCTTGTC TTTGAGACTG TCTATGTACA GTATGTGTAT AGAAGGACAT ATTTTACATT GATTATTTGT ATATTTTTAT AAACACATAA AGAGAGATAC -135
                                                                                                SP1
CGACGATGAC CAGGTTTATT ATCAGTTTCA TGACCATCAG TTTCTCCATG ACCTTTAACC CCTAATGAAG GCTGTGGGAA TGTCCCTCCT CTTTCTGCAC -35
                                                      PPARγ                            GC box
TGTAACTGAT CTGAGCTGGA GCCTTGGATT CTTCATTTAC ATCTTATCCA TACACACACA GACTCTTATC ATGGATATGG CTCTAATGAG CTTCCCAGAG +66
                                     TSS
AC +68
```

Figure S2. Nucleotide sequence of yellow catfish *cyp17α* promoter. Numbers are relative to the transcription start site (+1). The putative transcription factor binding

sites are underlined. The highlighted sequences show putative transcription factor binding sites.
